# Precision Oncology in Lower-Grade Gliomas: Promises and Pitfalls of Therapeutic Strategies Targeting IDH-Mutations

**DOI:** 10.3390/cancers14051125

**Published:** 2022-02-22

**Authors:** Pasquale Persico, Elena Lorenzi, Agnese Losurdo, Angelo Dipasquale, Antonio Di Muzio, Pierina Navarria, Federico Pessina, Letterio Salvatore Politi, Giuseppe Lombardi, Armando Santoro, Matteo Simonelli

**Affiliations:** 1 Department of Biomedical Sciences, Humanitas University, Via Rita Levi Montalcini 4, 20090 Milan, Italy; pasquale.persico@hunimed.eu (P.P.); angelo.dipasquale@humanitas.it (A.D.M.); antonio.dimuzio@humanitas.it (A.D.M.); federico.pessina@hunimed.eu (F.P.); letterio.politi@hunimed.eu (L.S.P.); armando.santoro@humanitas.it (A.S.); 2 Oncology Department, IRCCS Humanitas Research Hospital, Via Manzoni 56, 20089 Milan, Italy; elena.lorenzi@humanitas.it (E.L.); agnese.losurdo@humanitas.it (A.L.); 3 Radiotherapy Department, IRCCS Humanitas Research Hospital, Via Manzoni 56, 20089 Milan, Italy; pierina.navarria@humanitas.it; 4 Neurosurgery Department, IRCCS Humanitas Research Hospital, Via Manzoni 56, 20089 Milan, Italy; 5 Neuroradiology Department, IRCCS Humanitas Research Hospital, Via Manzoni 56, 20089 Milan, Italy; 6 Department of Oncology, Oncology 1, Veneto Institute of Oncology IOV-IRCCS, Via Gattamelata 64, 35128 Padua, Italy; giuseppe.lombardi@iov.veneto.it

**Keywords:** isocitrate dehydrogenase (IDH) mutations, lower-grade gliomas, IDH inhibitors, Glioblastoma, clinical trials, targeted therapy, precision oncology, vaccines

## Abstract

**Simple Summary:**

Mutations of isocitrate dehydrogenase (*IDH*) genes are the distinctive genetic feature of lower-grade gliomas (LGGs). Tumor-associated *IDH1/2* mutations result in a loss of normal enzymatic function and the abnormal production of 2-hydroxyglutarate (2-HG), which acts as an oncometabolite causing widespread changes in histone and DNA methylation and altering cellular metabolism. In the present review, we examine the “truncal” role of *IDH* mutations in gliomagenesis, giving hints on the different therapeutic strategies targeting *IDH1/2*-mutated gliomas. We analyze in detail, preclinical and, when available, data from clinical trials of specific inhibitors blocking the mutant enzyme, *IDH*-targeted immunotherapeutic approaches, and agents exploiting cellular metabolic and epigenetic vulnerabilities associated with the *IDH* mutant phenotype.

**Abstract:**

Mutations in isocitrate dehydrogenase (*IDH)1* and its homolog *IDH2* are considered an earliest “driver” genetic event during gliomagenesis, representing now the molecular hallmark of lower-grade gliomas (LGGs). *IDH*-mutated genes encode for a neomorphic enzyme that converts α-ketoglutarate to the oncometabolite D-2-hydroxyglutarate (2-HG), which accumulates to high concentrations and alters cellular epigenetics and metabolism. Targeting IDH mutations is the first attempt to apply “precision oncology” in LGGs. Two distinct strategies have been proposed so far and are under intense clinical investigation: (i) reducing the amount of intratumoral 2-HG by directly blocking the function of mutant IDH enzyme; (ii) exploiting the selective epigenetic and metabolic cellular vulnerabilities as a consequence of 2-HG accumulation. The present review describes the physiopathological mechanisms by which *IDH* mutations lead to tumorigenesis, discussing their prognostic significance and pivotal role in the gliomas diagnostic classification system. We critically review preclinical evidence and available clinical data of first-generation mutant-selective IDH inhibitors and novel IDH-targeted vaccines. Finally, as an alternative and attractive approach, we present the rationale to take advantage of selective 2-HG related epigenetic and metabolic weaknesses. The results of ongoing clinical trials will help us clarify the complex scenario of IDH-targeted therapeutic approaches in gliomas.

## 1. Introduction

Since the first discovery of isocitrate dehydrogenase (*IDH*) somatic mutations in 2006, major advances have been made in understanding their contribution to cancer development, providing a strong rationale for pharmacologically targeting the mutant metabolic enzyme.

The IDH family includes three different isozymes regulating key metabolic cellular processes, such as the ‘Krebs’ cycle, glutamine metabolism, lipogenesis, and redox balance. IDH1 is located in the cytosol and peroxisomes, while IDH2 and IDH3 in the mitochondrial matrix. Physiologically, IDH1-2 is responsible for the NADP-dependent oxidative decarboxylation of isocitrate to α-ketoglutarate (α-KG), producing NADPH in the process. Mutations of *IDH1* and *IDH2* have been identified in over 70% of lower-grade gliomas (LGGs; World Health Organization (WHO) grade II/III) and secondary glioblastoma (GBM) [1,2], and at lower frequencies in a variety of other human malignancies, including acute myeloid leukemia (AML; ≈30%), chondrosarcoma (≈50%), cholangiocarcinoma (≈15–20%), thyroid carcinoma, melanoma, angioimmunoblastic T-cell lymphoma, and in a rare subtype of breast cancer [3,4,5,6,7,8]. IDH3 is a heterodimer, not structurally related to the other two isoforms, and only rarely mutated in cancer.

Most common tumor-associated IDH1/2 mutations are located in key arginine residues (R132 for IDH1, R140, or R172 for IDH2) for the recognition of the substrate [9,10], and lead to a neomorphic gain of function enzyme with a markedly decreased affinity for isocitrate, capable instead of catalyzing the conversion of α-KG into 2-hydroxyglutarate (2-HG) in an NADPH-dependent manner. As a result, IDH-mutant (mut) cells accumulate 2-HG at supraphysiological levels (up to 100-fold higher than in IDH wild-type (wt) counterparts), while the intracellular concentration of α-KG greatly decreases [11].

It is now well documented that 2-HG has the properties of an “oncometabolite” contributing to tumorigenesis by altering epigenetic regulation, DNA repair mechanisms, multiple major metabolic pathways, and redox homeostasis [12,13].

**Figure 1:**
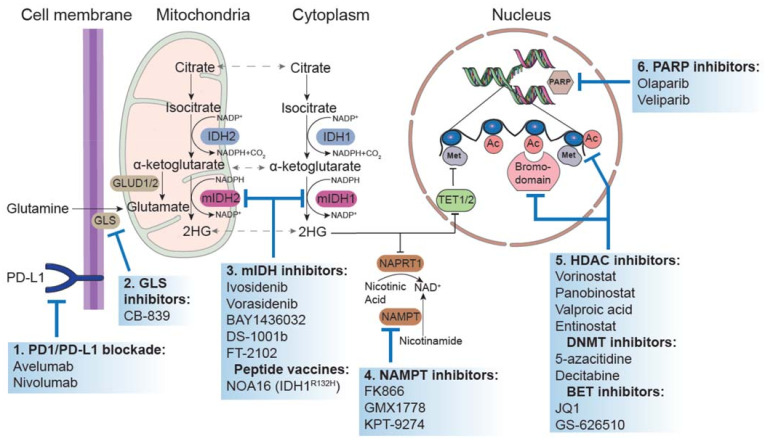
Investigational therapeutic approaches in IDH-mutant gliomas. (1) Many clinical trials are investigating the use of checkpoint inhibitors alone or in combinations with other interventions in IDH-mutated gliomas. (2) Glutaminase inhibitors could represent a potential therapeutic approach to lower 2-HG levels in IDH-mutated gliomas. (3) Both specific IDH inhibitors and IDH peptide vaccines have already shown encouraging efficacy in clinical trials. (4) IDH-mutated glioma cells are sensitive to the block of NAD^+^ biosynthesis through NAMPT inhibitors. (5) Epigenetic drugs capable of influencing transcriptomic profile and promoting tumor cell death are being developed as treatment for IDH-mutated gliomas. (6) As the accumulation of 2-HG induces homologous recombination DNA repair deficiency, PARP inhibitors alone or in combination with other interventions are under intense clinical investigation in IDH-mutated gliomas.

In the present review, we first explore the role of IDH mutations in gliomagenesis, discussing their prognostic significance and their crucial role in the evolution of the diagnostic classification system of glial tumors. We further discuss the preclinical rationale and emerging clinical data of all the different therapeutic approaches targeting IDH1/2-mut in gliomas, including inhibitors blocking the mut enzyme, immunotherapy, and agents exploiting cellular metabolic and epigenetic vulnerabilities associated with IDH-mut phenotype (Figure 1).

## 2. IDH Mutations in Gliomas

The identification of *IDH1/2* mutations in diffuse gliomas represents, doubtless, one of the major breakthroughs in the field of neuro-oncology over the last decades, which led brain tumors into a new molecular era, with several significant diagnostic, prognostic, and therapeutic implications.

Mutations in the *IDH1* gene were firstly observed in 2008 by Parsons et al. in a small subset (12%) of GBMs, mainly affecting young patients, progressed from previous low-grade tumors (secondary GBM), and carrying a much better outcome [2]. *IDH* mutations have been subsequently recognized as the distinctive genetic feature of LGGs, occurring in more than 90% of low-grade gliomas (WHO grade II) and in 70% of anaplastic gliomas (WHO grade III) [1]. In addition to peculiar clinical characteristics such as younger age at diagnosis and an overall better prognosis, accumulating evidence allowed identifying IDH-mutated gliomas as a completely different entity at the molecular level compared to the IDH-wt counterparts [14].

Nearly all 1p/19q codeleted gliomas (oligodendroglial tumors) harbor an *IDH1/2* mutation, along with O6-methylguanine-DNA methyltransferase (*MGMT)* promoter methylation and telomerase reverse transcriptase (*TERT*) promoter mutation, while *TP53* and *ATRX* alterations have been found in *IDH1/2*-mutated glioma without codeletion (astrocytic tumors). These great advances in molecular knowledge of brain tumors led, in 2016, to a major revision of the WHO classification with the introduction of the IDH mutational status and the presence of 1p/19q codeletion, in addition to histology, to define glioma subtypes [15]. Moreover, *IDH* mutations have been established as the most powerful positive prognostic factor for survival in gliomas, followed by age, tumor grade, and *MGMT* promoter methylation status. Although the underlying mechanisms have not yet been fully understood, the *IDH* mutational status seems to harbor also predictive significance, conferring an increased sensitivity to ionizing radiation and alkylating agents [16,17]. The WHO classification has been recently updated to better elucidate some gray areas. All IDH-mut diffuse astrocytic tumors have been considered as a single disease entity (astrocytoma, IDH-mut), graded (2, 3, or 4) based on the presence not only of microvascular proliferation or necrosis but also of other molecular markers with negative prognostic value, such as *CDKN2A/B* homozygous deletion. Conversely, IDH-wt low-grade astrocytoma harboring one or more of the three genetic features of classic GBM (*TERT* promoter mutation, epidermal growth factor receptor gene amplification, or combined gain of entire chromosome 7 and loss of entire chromosome 10), will be labeled as GBMs, carrying a very similar dismal prognosis [18].

In diffuse gliomas, over 90% of somatic *IDH* mutations are arginine-to-histidine heterozygous substitutions in the codon 132 (IDH1^R132H^ mutation) of the *IDH1* gene, with the remaining 10% represented by other amino acid substitutions located in the same spot. On the other hand, less than 1% of gliomas harbor *IDH2* hotspot mutations, mostly occurring at arginine 172 (IDH2^R172H^ mutation) or arginine 140 (IDH2^R140^ mutation) [19]. *IDH* mutations have been suggested to represent an early “truncal” event in the complex process of gliomagenesis, occurring in the stem cell progenitor from the subventricular zone stem cell niche before acquiring other “secondary” genetic alterations such 1p-19q codeletion, *ATRX*, and *TP53* mutations. The progressive accumulation of 2-HG over physiological levels impairs cellular metabolism, including glutamine catabolism and Krebs Cycle, and reshapes the epigenetics of the cell. The epigenetic alterations are thought to be an essential factor of gliomagenesis. 2-HG inhibits a wide range of a-KG-dependent dioxygenases, including lysine demethylases, Ten Eleven Translocase (TET) hydroxylases, Jumonji-C domain histone demethylases (JHDMs), and prolyl hydroxylase domain (PHD), leading to a global DNA and histone hypermethylation, stem-like cell differentiation block, and altered hypoxia-inducible factor (HIF) activity [20,21,22]. Consequently, IDH-mut gliomas acquire the CpG island methylator phenotype (CIMP or G-CIMP), which may be considered a sort of genetic signature of this class of tumors, with several transcriptional changes and the silencing of numerous loci in the genome. Based on the extent of global DNA methylation, IDH-mut/G-CIMP+ tumors have been further divided into two distinct subgroups characterized by different outcomes: G-CIMP-low gliomas with a low degree of DNA methylation and unfavorable clinical outcome and G-CIMP-high gliomas characterized by higher DNA methylation and better outcome [23,24]. It remains unclear whether *IDH* mutation by itself is responsible for tumor development or if it requires other oncogenic events to initiate gliomagenesis. Although DNA methylation is usually believed to be reversible, a study revealed that some of the DNA methylation sites in the IDH-mut cells might persist, even when the mutant enzyme is turned off [25]. This evidence supports the hypothesis that *IDH* mutation plays a key role in the first steps of malignant transformation, which becomes irreversible once the cells have initiated the oncogenic process [25]. However, emerging data suggest that for a subgroup of LGGs, 2-HG produced by the mutant enzyme may become non-essential for tumor progression as they acquire tertiary driver mutations and progress to a higher grade. *IDH* mutations would therefore behave as “passenger mutation,” losing their oncogenic function and even being eliminated in the latest stages of glioma progression [26].

## 3. IDH as Therapeutic Target for Treatment of IDH-Mutated Gliomas

It is not surprising that *IDH* mutations have been launched to the top of the list of new potential therapeutic targets for IDH-mut diffuse glioma. Two distinct strategies have been proposed so far and are currently under intense clinical investigation: (i) reducing the amount of intratumoral 2-HG by directly blocking the activity of the mutant IDH enzyme; (ii) taking advantage of the cellular vulnerabilities as a consequence of 2-HG accumulation. A key point that still needs to be addressed is how to locate an IDH-targeted therapy within the treatment paradigm of the IDH-mutated LGGs, which are typically slow-growing tumors arising in the young and displaying a good prognosis. LGGs’ therapeutic management consists of maximal safe surgical resection, eventually followed by radiotherapy and chemotherapy given as adjuvant for high-risk patients or postponed at recurrence/progression for low-risk cases. A crucial concern of neuro-oncologists in treating LGGs is the onset of long-term treatment-related side effects. In particular, radiation-induced neurocognitive defects can affect multiple domains, including memory, attentional and executive functioning, information processing speed, and problem-solving capability. These cognitive disorders caused by the exposure of healthy brain tissue, endocrine disorders, insomnia, and fatigue (related to the impairment of the hypothalamic-pituitary axis) may significantly compromise these young patients’ daily-life activities and social relationships. Furthermore, chemotherapy with alkylating agents may exacerbate the risk of developing long-term toxicities and accelerate malignant transformation. Thus, the development of novel therapeutic approaches in this setting, able to control tumor growth and high-grade progression, delaying or even avoiding the use of radiation and chemotherapy, clearly represents an urgent need.

Despite the great enthusiasm for what represents the first attempt of applying “precision oncology” to LGGs’ treatment, multiple observations call for some caution. Firstly, major epigenetic changes induced by the mut-IDH enzyme may persist even after suppressing 2-HG levels [25]. Secondly, blocking the mutant IDHs might, at least in theory, lead to the transformation of usually slow-growing and good prognosis tumors into more aggressive biological subtypes. Finally, once LGGs acquire other secondary and tertiary genetic alterations during their biological evolution, *IDH* mutations may lose their oncogenic function and even be eliminated at the latest stages of progression.

## 4. Targeting the Mutant Enzyme: IDH-Inhibitors

### 4.1. Rationale and Preclinical Studies

Chronologically, the development of specific IDH inhibitors represents the first approach evaluated in both IDH-mut hematologic and solid malignancies. The rationale for direct inhibition of the mutant enzyme relies on several observations: first, gliomas associated IDH mutations are located in hotspots within the enzyme’s active site; second, given their extremely high prevalence, the distinct DNA hypermethylation pattern and the persistence of IDH-mut clones, *IDH* mutations are considered an early oncogenic “driver” of LGGs; third, 2-HG showed in vitro similar cancer-promoting effects of the mutant IDH enzyme in a reversible fashion, emphasizing the pharmacological relevance of blocking the accumulation of this oncometabolite. However, the paucity of established preclinical models able to faithfully recapitulate the genetic landscape, tumor microenvironment, and growth patterns of IDH-mutated LGGs has made it extremely challenging for the investigation of the role of *IDH* mutations in glioma development and progression, and, consequently, of the therapeutic efficacy of IDH inhibitors.

The first evidence of activity of an IDH inhibitor in patient-derived glioma xenografts harboring IDH1^R132H^ mutation has been published by Rohle et al. in 2013 [27] (Table 1). Treatment of mice with the selective IDH1^R132H^ inhibitor AG-5198 led to marked reduction of intracellular 2-HG levels and delayed the growth of IDH1-mut TS603 glioma xenografts (* *p* = 0.015, two-tailed *t*-test), without impairing IDH1-wt TS516 xenografts [27]. Despite the evidence of histone H3K9^me3^ demethylation, no significant changes in global DNA methylation patterns have been observed. Moreover, mut-IDH1 inhibition suppressed clonogenicity, promoting the expression of genes associated with astroglial differentiation. However, these promising results were not reproducible later by other groups using different models. Indeed, despite a nearly complete depletion of intracellular 2-HG, no cell growth inhibition has been observed by Tateishi et al. when exposing eight endogenous IDH1-mut cell lines, including six IDH1^R132H^mut glioma tumor-initiating cells (TICs), HT1080 (IDH1^R132C^), and an IDH1^R132C^-mut melanoma (30T), to the same IDH1 inhibitor (AG-5198) [28] (Table 1). Continuous daily treatment with AG-5198 of an IDH1-mut recurrent GBM (MGG152) xenograft model in vivo did not affect mice survival, tumor size, and expression of the proliferative marker Ki-67 compared to controls [28]. In addition, prolonged exposure to this IDH1 inhibitor did not significantly modify the global trimethylation or dimethylation of histone H3K9, global trimethylation of histone H3K27, genome-wide patterns of DNA methylation, or the methylation of the *MGMT* gene promoter, leading to a paradox slight acceleration of cellular proliferation in vitro and significantly shorter survival of animals after transplantation [28].

On the other hand, Johannessen et al. demonstrated that early exposure to IDH1-mut inhibition suppressed 2-HG levels and blocked histone methylation (H3K4me3, H3K9me2, H3K9me3, or H3K27me3) of immortalized human astrocytes (IHAs) p53/pRb deficient [26]. However, when AG-5198 was administered just four days after the oncogenic insult, it had minimal effects on histone methylation and lost the ability to suppress clonogenicity in a time-dependent fashion [26].

The inconsistency of IDH inhibitors’ preclinical data in gliomas may depend on the fact that most IDH1-mut glioma cell lines and xenografts are derived from grade III or grade IV tumors with acquired tertiary genetic alterations in the mitogenic signaling pathways. These alterations may predominate over *IDH1* mutations as drivers, thus rendering the continued suppression of 2-HG non-essential during disease progression.

Due to its poor pharmacodynamics properties, precluding entrance into clinical trials, AGI-5198 has been replaced by AG-120 (ivosidenib), a highly specific and reversible inhibitor of IDH1-mut that competes for binding with the magnesium ion, a crucial enzymes’ cofactor, preventing the formation of a catalytically-active site [36]. AG-120 is characterized by a comparable selective potency across various IDH1^R132H^ mutants in different cell lines, without showing an inhibitory activity on wt or mut-IDH2 isoforms [29]. Despite a low rate of brain penetration in rats with an intact blood–brain–barrier (BBB), AG-120 showed a robust tumor and time-dependent 2-HG depletion in a mouse mut-IDH^R132H^ glioma (HT1080) xenograft model (IC50 range 5–13 nM) [29] (Table 1). Tumor 2-HG concentration declined rapidly, with maximum inhibition (92.0% and 95.2% at the 50 mg/kg and 150 mg/kg doses, respectively) achieved at ∼12 h post-dose [29]. Pharmacokinetic analysis revealed that AG-120 was detectable in the brain and brain tumor tissues of the mice, although at much lower exposures than in the plasma, indicating that AG-120 is highly potent against the mut-IDH1^R132H^ protein in vivo, leading to cellular differentiation and inhibition of cell migration and invasion [37].

The IDH1 pan-inhibitor BAY-1436032 slowed in vitro proliferation of primary glioma cultures, inducing their differentiation, with the astrocytic marker GFAP upregulated upon treatment. In two independent experimental series, BAY-1436032 has been shown to cross the BBB in vivo, significantly reducing 2-HG concentration (*p* = 0.00000057) and prolonging survival (*p* = 0.025) of intracranial glioma mut-IDH1^R132H^ xenografts compared to the untreated controls [30] (Table 1). However, similar to previous reports from subcutaneous and intracranial xenografts treated with AG-5198, no significant differences in genome-wide DNA methylation patterns nor promoter methylation of GFAP and SOX2 have been reported in these preclinical models [30].

AG-881 (vorasidenib) is an orally available, brain-penetrant, potent, small molecule inhibitor of IDH1 and IDH2-mut proteins. In a grade III IDH1-mut orthotopic patient-derived xenograft TS603 glioma model, vorasidenib treatment led to a >97% inhibition of 2-HG production in mice brains at doses ≥0.1 mg/kg. In addition, AG-881 also exhibits excellent brain penetration in vivo, with the brain-to-plasma area under the curve ratio (calculated using the total concentration of tumor plus brain tissue) being 1.33 and the brain tumor-to-plasma area under the curve ratio being 1.25 [31] (Table 1).

Kopinja et al. reported the discovery of a brain-penetrant IDH1-mut selective small molecule inhibitor (MRK-A) capable of potently inhibiting 2-HG production in two patient-derived orthotopic IDH1^R132H^-mut glioma xenograft models (BT142 and GB10) [38]. Despite the robust intracranial 2-HG inhibition observed in both mouse models, only BT142 displayed significant tumor growth inhibition, leading to a significant survival benefit, while in GB10 xenograft, such benefit was not observed [38]. Moreover, MKR-A treatment determined significant differences in the gene expression patterns between BT142 and GB10 tumors, with very few changes from baseline seen in the non-responsive GB10 model, despite a near-complete 2-HG inhibition [38]. Conversely, in MRK-A-treated BT142 tumors, a broad upregulation of gene expression has been observed, consistent with decreased levels of 5-methylcytosine and the reversal of DNA hypermethylation [38]. In particular, the authors reported a marked upregulation of the tumor suppressor gene CDKN1A/p27 in response to MRK-A treatment in BT142, suggesting that growth inhibition achieved in this model may be partly due to the elevated expression of the potent cell cycle inhibitor p27 [38]. The significant difference in responsiveness to IDH1-mut inhibition between the two orthotopic models used may also rely on the fact that BT142 produces much higher intra-tumoral 2-HG and expresses 4-fold higher levels of IDH-mut compared to GB10, suggesting that this latter model may no longer require 2-HG for maintaining its tumorigenic state.

In order to understand if the epigenetic reprogramming caused by *IDH*-mut is reversible, and, if so, what are the dynamics of reversibility, Turcan et al. used a multi-‘omics’ analysis of engineered IHAs and patient-derived glioma tumor spheres [25]. The authors found that *IDH1*-mut could generate a CD24+ population with a proliferative advantage and stem-like transcriptional features [25]. However, methylation changes upon loss of *IDH1*-mut were generally slow, requiring many cell divisions to revert to baseline levels [25]. Moreover, prolonged exposure to *IDH1*-mut led to irreversible genomic and epigenetic alterations with a subset of IDH1^R132H^-induced methylated loci that persisted even after suppressing mut-IDH1 expression [25]. Moure et al., using patient-derived IDH1^R132H/WT^ glioma cell lines and CRISPR-Cas9-mediated gene knockout, showed that abolishing of 2-HG production by genetic deletion of IDH alleles is not sufficient to completely reverse hypermethylation of all G-CIMP loci, despite a widespread pattern of demethylation and the formation of a G-CIMP-low-like phenotype [39].

### 4.2. Clinical Studies

#### 4.2.1. Ivosidenib

Safety, tolerability, and preliminary activity of ivosidenib in advanced IDH1-mut solid tumors were investigated in a multicenter Phase I study (NCT02073994), comprising a dose escalation and a dose-expansion part. Overall, the trial included patients with cholangiocarcinoma, chondrosarcoma, and 66 patients affected by recurrent IDH1-mut gliomas (thirty-two grade II, eighteen grade III, twelve grade IV, four with unknown grade), that had received a median of two previous systemic therapies (range 1–6), including Temozolomide, Nitrosureas, and Bevacizumab [40] (Table 2). No dose-limiting toxicities (DLTs) were reported in the dose-escalation phase, and the maximum tolerated dose (MTD) was not reached [40]. Based on the pharmacokinetic/pharmacodynamic data, safety profile, and preliminary clinical activity, a dose of 500 mg once daily was selected for the expansion part of the trial [40]. Considering the association between contrast-enhancement pattern and glioma transformation and progression, patients with glioma were divided into two groups according to baseline MRI: non-enhancing (35 cases) versus enhancing lesions (31 cases) [40]. Any grade adverse events (AEs) were observed in 63 of 66 patients (95.5%), most common being headache (39.4%), fatigue (22.7%), nausea (22.7%), and vomiting (19.7%) [40]. Overall, 13 out of 66 experienced severe (grade ≥ 3) AEs, including headache (4.5%), hypophosphatemia (3%), and seizure (3%) [40]; 39 out of 66 patients (59.1%) experienced treatment-related AEs (TRAEs) that were mostly mild to moderate in severity and easily manageable [40]. Severe TRAEs were reported only in 2 patients (neutropenia, decreased weight, hyponatremia, and arthralgia) [40]. No patients required dose reductions or discontinued treatment due to AEs, while a dose interruption because of an AE occurred in eight cases (12.1%) [40].

Among the 66 patients treated in both dose escalation and dose-expansion phases (1 at the dose of 100 mg BID, 6 at 300 mg QD, 50 at 500 mg QD, 5 at 600 mg QD, and 4 at 900 mg QD) ([40], Supplementary Appendix), according to the investigator’s assessment, one achieved a partial response (PR), 44 patients showed stable disease (SD), while progressive disease (PD) occurred in 21 cases [40]. SD was achieved in 85.7% of cases in the non-enhancing cohort, with a median progression-free survival (PFS) of 13.6 months [40]. The 6-months of tumor volume growth rate (TvGR) in this group was 26% before study entry treatment and drastically decreased to 9% under treatment with ivosidenib [40]. In the enhancing cohort, the disease control rate (DCR) resulted lower (45.2%), with a poor median PFS of only 1.4 months [40]. These data seem to suggest that ivosidenib may exert its anticancer activity predominantly against non-enhancing gliomas.

As already mentioned, a possible explanation for such results is that non-enhancing gliomas represent an earlier form, where the role of IDH mutations for tumor growth is still crucial, while enhancing “transformed” IDH-mutated LGGs are driven by the acquisition of additional mutations, with limited susceptibility to therapeutic reversion of epigenetic changes induced by IDH-mut. Supporting this hypothesis, the presence of genetic alterations in cell cycle genes was associated with shorter PFS even in non-enhancing gliomas [40].

#### 4.2.2. Vorasidenib

Vorasidenib (AG-881) was the first-in-class oral dual inhibitor of both IDH1-mut and IDH2-mut, with an effective brain penetrance, to enter clinical development. Dual inhibition of IDH1/2-mut may be superior to isoform-selective inhibition because isoform switching from IDH1-mut to IDH2-mut, or vice versa, has been reported as a potential mechanism of acquired resistance in AML.

Results from a multicenter dose-escalation Phase I study designed to assess the safety and tolerability of vorasidenib in advanced IDH1/2-mut solid tumors were recently published (NCT02481154) (Table 2). Investigator-assessed objective response was determined according to RANO criteria for enhancing gliomas and RANO-LGGs’ criteria for non-enhancing gliomas. In the latter case, minor responses (25–50% reduction in T2/FLAIR tumor) were included in the definition of objective response [41].

Overall, 93 patients with IDH1/2-mut advanced solid tumors were enrolled, of whom 52 patients had a diagnosis of recurrent glioma (22 with a non-enhancing disease, 30 with enhancing disease) [41]. Twenty-five cases (48.1%) were grade II tumors, and 22 (42.3%) grade III tumors, mostly harboring a mutation in the IDH1 gene (92.3%) [41]. Thirty-nine (75%) patients received prior systemic therapies (median number 2, range 1–6) [41]. At the analysis cutoff date (29 April 2020), 8 (36.4%) patients within the non-enhancing group remained on treatment, with 10 (45.5%) discontinuing treatment due to progression, 2 (9.1%) due to AEs, and 2 (9.1%) withdrawing the consent [41]. In the overall population, DLTs (grade ≥ 2 transaminases elevations) occurred in 5 patients at ≥100 mg dose levels, leading to treatment discontinuation in 2 cases [41]. Increased transaminase levels were dose-dependent, not associated with a bilirubin elevation, and usually resolved to grade ≤1 with dose modification or interruption [41]. Though MTD/RP2D was not reached according to the Bayesian model adopted, the clinical team recommended no further escalation beyond 300 mg and thus to proceed with doses <100 mg in glioma patients [41]. All-grade TRAEs were reported in 38 (73.1%) patients with glioma, including 4 of grade ≥3; 2 (3.8%) patients discontinued treatment due to AEs, and 7 (13.5%) required a dose reduction due to AEs [41].

In the non-enhancing glioma group (*n* = 22), the overall response rate (ORR) was 18% (1 PR, three minor responses), while 17 (72.7%) patients had SD as the best response [41]. All responses were of noteworthy duration, ranging from 7.4 to 27.7 months [41]. Conversely, no patients with enhancing gliomas had a confirmed objective response, with 17 out of 30 (56.7%) patients achieving SD as their best response. The median treatment duration was 26.8 months (range: 1.0–50.9) for non-enhancing glioma and 3.3 months (range: 0.2–53.6) for enhancing glioma [41]. Fifteen (68.2%) patients with non-enhancing tumors and 4 (13.3%) with enhancing tumors received study treatment for >1 year [41]. In the overall glioma population, median PFS was 7.5 months, 36.8, and 3.6 months in non-enhancing and enhancing groups, respectively [41].

The promising, albeit preliminary, signs of efficacy seen in the non-enhancing cohort led to the design of the INDIGO study, a Phase III trial investigating the role of vorasidenib 50 mg QD in patients with residual or recurrent IDH1/2-mut grade 2 glioma, who are under radiographic surveillance following surgery and have not yet received radiation or chemotherapy (NCT04164901) (Table 3).

#### 4.2.3. Ivosidenib and Vorasidenib in Recurrent Low-Grade Glioma

Ivosidenib (AG-120) and vorasidenib (AG-188) are currently under investigation also in a Phase I trial enrolling patients with recurrent non-enhancing LGGs with IDH1^R132H^ mutation who will undergo surgery (NCT03343197) (Table 2). The goal of this study is to evaluate 2-HG suppression in brain tumor patients following four weeks of treatment with AG-120 (*n* = 13) or AG-188 (*n* = 14) compared to untreated controls [42]. Overall, 27 patients received either ivosidenib or vorasidenib, and 2-HG concentration was 92% and 92.5% lower in resected tumor tissue of treated patients, respectively, indicating near-complete inhibition of the enzyme [42]. Common (≥4 patients) all-grades of TEAEs included diarrhea (37%), hypocalcemia, constipation, and nausea (each 18.5%), anemia, hyperglycemia, pruritus, headache, and fatigue (each 14.8%) [42]. Interestingly, as of June 2019, only one patient receiving the study drugs experienced PD and discontinued treatment at the time of analysis [42]. Updated data presented at ASCO Annual Meeting 2021 revealed that an optimal 2-HG suppression is associated with upregulation of neural differentiation-related gene expression, downregulation of stemness-related gene expression, decreased tumor proliferation, increased T-cell infiltration and upregulation of type I interferon signaling and antigen presentation related-genes [46].

#### 4.2.4. BAY-1436032

Wick and colleagues reported a multicenter, first-in-human, Phase I dose-escalation trial aimed to determine the safety and tolerability of BAY-1436032 in patients with IDH1^R132X^-mut advanced solid tumors (NCT02746081) (Table 2). The study included a dose-escalation phase to determine MTD and recommended Phase II dose (RP2D), followed by dose-expansion cohorts to explore further the safety and clinical efficacy of BAY-1436032 in specific tumor types (LGG, GBM, intrahepatic cholangiocarcinoma, and a basket cohort of other tumor types). Overall, 55 glioma patients were treated in the trial, 17 (14 LGGs and 3 GBMs) into the dose-escalation phase, and 38 (25 LGGs and 13 GBMs) into the dose-expansion [43]. Of note, about 75% of LGG cases had anaplastic grade 3 tumors, and most of them (33 out of 35) had contrast-enhancing lesions, as this was an eligibility criterion to be enrolled in the dose-expansion [43].

In the dose-escalation part, TEAEs related to BAY-1436032 occurred in 11/29 patients (38%); all were grade ≤2, and no one led to dose modification or drug discontinuation; no DLT occurred, and the MTD was not reached [43]. Therefore, the dosage of 1500 mg twice daily was selected for expansion cohorts based on PK data [43]. In the second part of the study, 6/52 patients (12%) experienced a grade ≥3 TEAE deemed to be related to the study drug, one of which was grade ≥4 (grade 4 lipase increase); 12/52 (31%) needed dose reductions, and 9/52 (24%) had to discontinue treatment because of TEAEs [43].

Overall, among the 35 LGGs evaluable patients for efficacy across all dose levels, 1 (3%) achieved CR, 3 (9%) PR, and 15 (43%) SD as the best response, with a PFS rate at three months of 0.31 (0.15–0.46) [43]. Conversely, among the 14 evaluable GBM patients, none achieved an objective response (29% SD), and the PFS rate at three months was 0.22 (0–0.44) [43]. At the time of data cutoff (August 2020), only patients with LGG were still on active treatment (one with CR, one with PR, and two with SD) [43].

#### 4.2.5. DS-1001b

DS-1001b is an oral selective inhibitor of mutant IDH1^R132X^ designed to penetrate the BBB. Preliminary results of the first-in-human, multicenter, phase I study (NCT03030066) investigating the safety of DS-1001b in 45 patients with a diagnosis of recurrent/progressive IDH1-mut glioma were presented at the ASCO annual meeting 2019 (Table 2). The most frequent AEs (>20%) were skin hyperpigmentation, diarrhea, pruritus, nausea, rash, and headache [44]. No drug-related severe AEs occurred, only one DLT was reported (grade 3 white blood cell count decreased) at the dose of 1000 mg twice daily, and the MTD was not reached [44]. Among 29 evaluable patients with contrast-enhancing gliomas, 1 achieved CR, 3 PR, and 10 SD, while among nine evaluable patients with non-enhancing gliomas, 2 achieved a minor response and 7 SD [44]. A phase II study in patients with chemotherapy and radiotherapy naïve IDH1-mutated grade 2 glioma is ongoing (NCT04458272) (Table 3).

## 5. Immunotherapeutic Approaches for IDH Mutant Gliomas

### 5.1. Preclinical Studies

Given the remarkable clinical successes in other tumor types, great efforts have been undertaken in latest years to explore the role of immunotherapy in the field of neuro-oncology and even in the context of LGGs. It has been observed that spontaneous IDH1^R132H^-specific CD4+ T-helper-1 (TH1) and humoral immune responses might occur in LGG patients. Moreover, there are now several preclinical evidences demonstrating that the oncometabolite 2-HG greatly contributes to shaping the immunosuppressive glioma microenvironment and plays a crucial role in immune escape mechanisms [47]. Berghoff et al., analyzing a cohort of 43 WHO grade II/III gliomas (39 IDH-mut, 4 IDH-wt) and 14 IDH-mut GBMs, demonstrated that IDH-mut tumors have fewer CD3+ and PD1+ tumor-infiltrating lymphocytes (TILs) and decreased expression of programmed cell death ligand (PD-L1) compared to their wt counterparts (series of 117 IDH-wt GBMs) [48]. Using data of 677 diffuse gliomas grades II-IV from The Cancer Genome Atlas (TCGA) database, authors found that PD-L1 gene expression was statistically significantly higher in IDH-wt WHO grade II/III gliomas compared with IDH-mut WHO grade II/III gliomas [48]. Moreover, PD-L1 gene promoter methylation levels were higher in IDH-mut than IDH-wt samples [48].

Gene expression data from the same TCGA database revealed a reduced expression of cytotoxic T lymphocyte-associated genes and IFN-γ-inducible chemokines, including CXCL10 in IDH-mut tumors compared with IDH-wt tumors [49]. The introduction of mutant IDH1 or treatment with 2-HG in immortalized normal human astrocytes and syngeneic mouse glioma models led to a reduction of CXCL10 levels, which was associated with decreased production of STAT1 and also suppressed the accumulation of T-cells into tumors [49]. All these effects are reversible by treatment with IDH-C35, a specific inhibitor of IDH1-mut.

Bunse et al. demonstrated that 2-HG directly impairs T-cells’ activation, proliferation, and cytokine secretion by altering the calcium-dependent transcriptional activity of nuclear factors and suppressing ATP-dependent TCR signaling and polyamine biosynthesis [32]. Moreover, in different syngeneic IDH1-mut tumor models, 2-HG inhibited the development of antitumor T-cell immunity induced by IDH-1 vaccination, adoptive T-cell transfer, and checkpoint blockade while pharmacological inhibition of the neomorphic enzymatic function of mut-IDH1 by administration of BAY-1436032 alleviates intratumoral immune suppression [32] (Table 1).

Zhang et al. have shown that glioma cells harboring IDH1 mutation acquire resistance to natural killer (NK) cell-mediated lysis due to the epigenetic silencing of NKG2D ligands ULBP1 and ULBP3 by 2-HG-induced hypermethylation [50], making IDH-mut tumors less vulnerable to NK-cell-mediated lysis as compared to IDH-wt. Furthermore, decitabine-mediated hypomethylation restores ULBP1 and ULBP3 expression in IDH-mut glioma cells, suggesting a potential method to sensitize IDH-mut gliomas to NK cell-mediated immune surveillance in the clinic [50].

A couple of years later, the same group demonstrated that 2-HG impaired both the classical and the alternative pathways of complement activation, provoking a reduced complement-mediated glioma cell injury and a decreased complement-mediated opsonization and phagocytosis [51]. Moreover, 2-HG inhibits antitumor T-cell response, directly suppressing T-cell migration, proliferation, and cytokine secretion [51].

IDH1^R132H^ is a tumor-specific neoantigen with high penetrance and uniform expression in all tumor cells, representing an ideal target for mutation-specific vaccination strategies. An immunogenic epitope in the IDH1^R132H^ protein suitable for building a mut-specific vaccine has been firstly identified by Schumacher and colleagues [33]. In a murine sarcoma model lacking mouse MHC and transgenic for human MHC class I and II, vaccination with IDH1^R132H^ p123–142 resulted in robust interferon (IFN)-γ mutation-specific T-cell responses that were effective in control growth of syngeneic IDH1(R132H)-expressing tumors [33] (Table 1). IDH1-mut vaccines demonstrated efficacy also in an intracranial glioma model genetically modified to harbor the R132H mutation, producing a significant survival gain compared to controls and 25% of cured mice [34] (Table 1). In addition, higher amounts of peripheral CD8+ T-cells, higher production of IFN-γ, and evidence of anti-IDH1-mut antibodies were observed in immunized mice [34].

More recently, Kadiyala et al. developed a genetically engineered mouse model of glioma expressing IDH1^R132H^ and loss of ATRX and TP53 to better understand the role played by 2-HG in influencing the glioma immune microenvironment [35] (Table 1). 2-HG inhibition by treatment with a specific IDH1^R132H^ inhibitor alone or in combination with radiation and temozolomide (TMZ) substantially prolonged survival of IDH1-mut glioma-bearing mice, increased PD-L1 expression levels to similar levels as observed in IDH-wt gliomas, contributing in also generating an anti-glioma immunity [35]. Moreover, the coadministration of PD-L1 blockade with IDH1^R132H^ inhibition and the standard of care (RT + TMZ) markedly enhanced the outcome of IDH1-mut glioma-bearing mice, counteracting T-cell exhaustion and eliciting an immunological memory with the generation of memory CD8+ T-cells [35]. These data supported the design of clinical trials investigating the efficacy of IDH1^R132H^ inhibitors in combination with standard of care (SOC) and anti-PD-L1 immune checkpoint blockade to treat glioma patients expressing with IDH mutations.

### 5.2. Clinical Trials

Based on all these preclinical data, the German National Cancer Center has conducted a multicenter, first-in-humans Phase I trial, evaluating the feasibility, safety, and immunogenicity of a vaccine targeting the IDH1^R132H^ mutant protein among newly diagnosed patients with IDH1-mut glioma (NOA16, NCT02454634) [45,52] (Table 2).

The vaccine consisted of 300 mg of a 20-mer R132H peptide emulsified in montanide and was administered subcutaneously at 2-week intervals for the first four doses, followed by four additional doses given every four weeks (weeks 1, 3, 5, 7, 11, 15, 19, and 23). The primary safety endpoint was the Regime-Limiting Toxicity (RLT), defined as protocol-specified, treatment-related, severe AEs [45].

Immunogenicity was evaluated by assessing an IDH1^R132H^-specific T and/or B-cell response, measured by IFN-γ ELISpot and ELISA, respectively, at any established time points (six in total) during the trial. Brain MRI scans were performed every three months to assess disease response according to the RANO criteria [45].

From May 2015 to November 2018, a total of 33 patients with histologically-confirmed IDH1^R132H^-mutated, newly-diagnosed grade 3 or 4 astrocytomas, according to the WHO 2016 classification, were enrolled across seven German centers, and 32 of them received at least one dose of vaccine [45]. Patients were divided into three subgroups, according to their previous treatments: RT alone (treatment group 1, including six patients), CT with TMZ for three cycles (treatment group 2, including three patients), or standard chemoradiation with TMZ (treatment group 3, including 23 patients) [45]. Vaccination for patients in group 1 started 4–6 weeks after the end of radiotherapy, for patients in group 2 on the fourth cycle of TMZ monotherapy, and for patients in group 3 on the first cycle of adjuvant TMZ, respectively [45]. About two-thirds (66%) of the patients had grade 3 astrocytoma, while 34% had a grade 4 tumor [45]. A gross total resection was performed in about half of the cases (53%), while 38% and 9% of patients underwent a subtotal resection or just a biopsy, respectively [45].

The trial met its primary safety endpoint as IDH1^R132H^-vaccine was well tolerated, with no RLTs observed, and most of the AEs consisting of mild site reactions; 29 out of the 32 patients completed all planned study vaccinations [45].

Most patients (93.3%) developed an IDH vaccine-induced immune response; T-cell responses were observed in 26 out of 30 patients analyzed, while B-cell responses in 28 out of 30, respectively [45]. A mutation-specificity score (MSS) was built as an explorative measure of the magnitude and duration of IDH1^R132H^-induced T-cell responses: it was observed that higher MSSs were associated with high levels of TH1 and TH17 T-helper cell subtypes cytokines, such as IL-17, TNF, and IFN-gamma [45].

With a median follow-up of 46.9 months, PFS and OS at three years were 63% and 84%, respectively, with no significant differences between grade III and grade IV patients [45].

The authors described a relatively high rate of pseudoprogression in the trial population (37.5%, 12 out of 32 patients) compared to that reported in a molecularly-matched 60-patient population selected as a control cohort (16.5%, 10 out of 60 patients) [45]. Pseudoprogression was observed only in patients with a detectable immune response in the peripheral blood and correlated with IDH vaccine-specific T-cell levels [45]. Only one patient with PsPD underwent post-vaccination surgery, and the presence of IDH1 R132H-reactive intratumoral CD4+ TCR14+ cells was confirmed in the surgical specimen, suggesting that vaccination induces a clonal expansion of specific T-helper cells able to reach the brain tissue [45].

Mature clinical data of immune checkpoint blockade in IDH-mut gliomas are still lacking. However, a Phase II clinical trial (NCT02968940) in which the anti-PD-L1 avelumab has been associated with hypofractionated RT as treatment of patients with IDH-mut GBMs recently completed the enrollment, and results are now awaited (Table 3). In addition, there are many other clinical trials investigating PD-1/PD-L1 blockade alone or in combination with other agents, such as specific mut-IDH inhibitors or PARP inhibitors (NCT03991832, NCT03557359, NCT03718767, NCT03925246), still ongoing. Results of these studies, when available, will help clinicians to clarify the role of checkpoint inhibition in the context of IDH-mut LGGs (Table 3).

## 6. Targeting IDH-Mutant-Associated Epigenetic and Metabolic Vulnerabilities

An alternative therapeutic strategy consists in exploiting, rather than reverting, the IDH-mut phenotype targeting the cellular epigenetic and metabolic vulnerabilities associated with the IDH mutations that persist, even at later stages of tumor development.

### 6.1. Targeting the DNA Damage Pathway

Among the most significant cellular weaknesses derived from mutations in the *IDH* gene exploitable with therapeutic intent are defects in the DNA repair processes.

It has been now well-established that accumulation of 2-HG induces homologous recombination (HR) DNA repair deficiency by influencing histone methylation and establishing a “BRCAness” phenotype [53]. In that file, the pioneering work by Sulkowski et al. in 2017 suggested that 2-HG-induced HR suppression derived primarily by direct inhibition of ɑ-KG-dependent dioxygenases, particularly KDM4A and KDM4B [54]. Moreover, this “BRCAness” phenotype renders primary patient-derived glioma cells ex vivo and genetically-matched tumor xenografts in vivo, highly sensitive to poly(adenosine 5′-diphosphate) ribose polymerase (PARP) inhibition (olaparib). Conversely, HR defects may be reversed using IDH1-mut inhibitors, AGI-5198, AG-120, and IDH1-C227 [54]. According to Lu et al., an alternative explanation for the impairment of PARP-mediated DNA repair pathway in IDH-mut cells is the compromised oxidative metabolism and decreased NAD^+^ availability, explaining the peculiar chemosensitivity of IDH-mut gliomas in vivo [55].

Results of subsequent preclinical studies indicated that the efficacy of PARP inhibition in IDH-mut tumors might be further enhanced by the combination with TMZ or RT [56,57]. Using multiple in vitro and preclinical animal models of glioma and cholangiocarcinoma, Wang et al. demonstrated that the activity of PARP inhibitor veliparib had been synergistically enhanced by concurrent, localized radiation therapy, while the same radiosensitizing effect was not observed in IDH-wt xenografts [56]. Higuchi et al., using isogenic pairs of glioma cells with or without RNAi-mediated MSH6 deficiency, found that PARP inhibition alone did not significantly influence cell viability, independently of IDH and MSH6 status, whereas it was sufficient to restore TMZ chemosensitivity in MSH6-inactivated glioma [57].

Based on all these preclinical observations, the use of PARP inhibitors, alone (NCT03212274 and NCT03561870) or in combination with TMZ/RT (NCT03749187 and NCT03914742) or checkpoint inhibitors (NCT03991832), is currently under intense clinical investigation (Table 3).

### 6.2. Metabolic Vulnerabilities

#### 6.2.1. NAD^+^ Metabolism

NAD+ is a crucial cofactor used in many essential cellular metabolic pathways. Systematically investigating metabolic vulnerabilities of IDH-mut cancers, Tateishi et al. discovered that these tumors are exquisitely susceptible to depletion of the canonical coenzyme NAD^+^ [28]. NAD^+^ depletion activated the intracellular energy sensor AMPK, triggered autophagy, and resulted in cytotoxicity [28].

IDH1-mut glioma cells lowered NAD^+^ levels by silencing the NAD^+^ salvage enzyme nicotinic acid phosphoribosyltransferase (NAPRT) and are sensitive to the block of NAD^+^ biosynthesis via nicotinamide phosphoribosyltransferase (NAMPT) inhibition [28]. Both the NAMPT inhibitors FK866 and GMX1778, potently inhibited cell viability of six endogenous IDH1/2-mut cancer cell lines, including three IDH1^R132H^-mut GBM TICs (MGG119, MGG152, and BT142), the IDH1^R132C^-mut lines HT1080 and 30T, SW1353 chondrosarcoma (IDH2^R172S^), with IC_50_ values ranging between 1 and 25 nM. In contrast, 8 IDH1/2-wt cancer cell lines derived from similar tissue types and normal human astrocytes (NHAs) were resistant at doses up to 10 mM, indicative of a wide therapeutic index [28].

Using patient-derived IDH-mut xenografts, the same group showed that combining NAMPT inhibitors with the DNA-alkylating agent TMZ increased cytotoxic effects on IDH-mut cells by increasing NAD^+^ consumption and prolonged mouse survival when compared to each agent used alone [58].

Multiple NAMPT inhibitors were developed, and one of these (KPT-9274) is now being tested in clinical trials (NCT02702492) (Table 3).

#### 6.2.2. Mitochondrial Metabolism and Oxidative Stress

A synthetic lethal interaction between mutant IDH1/2 and members of the BCL-2 family was first reported in AML tumor models [59]. IDH2-mut primary human AML cells were more sensitive to ABT-199, a highly specific BCL-2 inhibitor, both ex vivo and in xenotransplant models [59]. This sensitization effect was induced by 2-HG-mediated inhibition of the activity of cytochrome c oxidase (COX) in the mitochondrial electron transport chain (ETC); suppression of COX activity lowered the mitochondrial threshold to trigger apoptosis upon BCL-2 inhibition [59].

Solid tumors seem to be more often dependent on BCL-xL for their survival. In six model systems, including patient-derived stem cell-like GBM cultures, inhibition of Bcl-xL induced significantly more apoptosis in IDH1-mut cells than in IDH1-wt cells [60]. In an orthotopic GBM xenograft model expressing mutated IDH1, Bcl-xL inhibition leads to long-term survival [60].

#### 6.2.3. Amino Acid Metabolism (Glutamate/Glutamine/Glucose)

Glutamine and glutamate are amino acids that play a significant role in cell metabolism as major sources of carbon and nitrogen. Most of the glutamine is converted to glutamate by the glutaminase (GLS) to be used in many different metabolic reactions, particularly for the synthesis of glutathione, which is an important antioxidant, or converted to α-KG either by glutamate dehydrogenases (GLUDs) or transaminases (BCAT1 and BCAT2). IDH-mut cells use α-KG, a product of glutaminolysis, to produce 2-HG, suggesting GLS inhibition as a potential therapeutic approach to lower 2-HG levels. The two enzymes converting glutamate to ɑ-KG, GLUD1, and GLUD2, are overexpressed in IDH1-mut tumors, and orthotopic growth of an IDH1-mut glioma line was inhibited by knockdown of GLUD1/2 [61]. Inhibiting glutaminase (GLS) activity by siRNA or the small molecule inhibitor bis-2-(5-phenylacetamido-1,2,4-thiadiazol-2-yl)ethyl sulfide (BPTES) slowed the growth of GBM cells expressing IDH1-mut [61].

2-HG potently inhibits the ɑ-KG-dependent transaminases BCAT1 and BCAT2, thereby decreasing glutamate levels and increasing dependence on GLS for the biosynthesis of glutamate and one of its products, glutathione [12]. Inhibiting GLS in IDH1-mut glioma cells, in which 2-HG already suppresses BCAT, significantly impairs their fitness by lowering glutamate, glutathione, and resistance to oxidative stress in vitro and to radiation in vitro and in vivo [12].

## 7. Notch Pathway

The cell surface Notch ligand delta-like 3 (DLL3) has recently emerged as a therapeutic target in cancer [62]. DLL3 inhibits Notch pathway activation in cis and in trans by redirecting or retaining Notch and activating ligand DLL1 to late endosomal/lysosomal compartments or the Golgi, preventing their localization to the cell surface [63].

Spino et al. found that IDH-mut gliomas have significantly higher Notch ligand delta-like 3 (DLL3) RNA (*p* < 1 × 10^−15^) and protein by immunohistochemistry (IHC) (*p* = 0.0014 and *p* < 4.3 × 10^−6^), whereas expression was patchy, low, or absent in IDH-wt GBM [64]. In addition, DLL3 expression was intense, homogeneous, and retained in all recurrent tumors [64], while it was rarely detected by IHC in nontumor brain samples being considered as a tumor-associated antigen. Moreover, patient-derived IDH-mut glioma tumor spheres overexpressing DLL3 were potently and selectively sensitive to anti-DLL3 antibody rovalpituzumab tesirine (Rova-T) in an antigen-dependent manner [64].

## 8. Epigenetic Approaches

### 8.1. DNA Methyltransferase Inhibitors (DNMTi)

Given the well-described hypermethylated phenotype hallmark of IDH-mut malignancies, DNA demethylating agents, capable of inducing long-term epigenetic reprogramming, were among the first therapeutic approaches investigated for treating those cancers. In preclinical models of IDH1-mut gliomas, long-term administration of DNA methyltransferase inhibitors (DNMTi), 5-azacitidine and decitabine, induced reduction of DNA methylation, glial differentiation, and a significant tumor growth inhibition in xenograft models [65,66]. The antitumor effect of azacitidine was enhanced when combined with TMZ in both subcutaneous and orthotopic xenograft models of IDH1^R132H^-mut glioma, with mice treated with the combination showing a significant increased survival [67].

Despite these promising preclinical results, the first clinical use of azacitidine given as therapy of 12 heavily pretreated patients with IDH1/2-mut recurrent gliomas with astrocytic or oligodendroglial histology showed only minimal activity: no patient achieved a radiographic response, while five (41.7%) had disease stabilization, of whom 2 (16.7%) lasting for more than 18 months [68]. Other clinical trials, testing 5-azacytidine as single agent or in combination with IDH-mut inhibitors (Olutasidenib), are now ongoing (NCT03666559, NCT03684811) (Table 3). Another Phase I study is exploring the safety and activity of ASTX727, a novel agent that combines decitabine with a cytidine deaminase inhibitor cedazuridine in recurrent/progressive non-enhancing IDH-mut gliomas (NCT03922555) (Table 3).

### 8.2. Histone Deacetylse Inhbitors (HDACi)

Histone deacetylase inhibitors (HDACi), such as vorinostat, panobinostat, valproic acid (VPA), and entinostat, represent another emerging class of epigenetic drugs capable of influencing transcriptomic profile and promoting tumor cell death. For example, Elahi et al. found that VPA (an HDACi and antiepileptic drug) inhibited the growth of patient-derived IDH1-mut glioma cell lines, and both VPA and panobinostat (BH589) upregulated similar gene sets promoting expression of several previously repressed genes [69].

### 8.3. Bromodomain and Extra-Terminal Motif (BET) Inhibitors

Another promising target for treating IDH-mut gliomas is the bromodomain and extra-terminal motif (BET) proteins, which play a pivotal role in epigenetic regulation, particularly promoting the high-level expression of oncogenes. Blocking the bromodomain protein BRD4 has been shown to induce rapid differentiation and cell death in IDH2-mut AML mice models through the reduction of levels of MYC [70]. In six patient-derived IDH1-mut primary glioma cultures, IDH1-mut glioma cells have proven to be very sensitive to two different BET inhibitors in vitro (the well-established JQ1 compound and GS-626510), with an IC50 value less than 250 nM and achieving maximum cell death of 31 to 77% [71].

Table 1 summarizes the main preclinical studies of IDH-targeted agents in glioma mouse models, Table 2 shows the clinical trials with available data so far, while in Table 3 are presented the main ongoing clinical studies for IDH-mutant gliomas.

## 9. Conclusions and Future Directions

Identifying *IDH* mutations as the distinctive genetic hallmark of LGGs has determined significant changes in the diagnostic and prognostic management of these tumors, paving the way for the first time to different targeted therapeutic opportunities. *IDH* mutations have now been widely recognized as a truncal event occurring early during the gliomagenesis, with the 2-HG-associated epigenetic impact and block of differentiation playing a key role in driving the entire oncogenic process. Compared to IDH-wt GBM, IDH-mut LGGs affect younger patients, have a significantly lower growth rate carrying a much better prognosis, and generally do not show contrast enhancement on T1-weighted brain MRI at initial diagnosis. The current treatment paradigm of LGGs includes maximal safe tumor resection, followed by radiation and CT when indicated. Unfortunately, most patients suffer disease recurrence and progress to a higher-grade tumor. Even in the case of long-lasting disease control, patients often experience a significant worsening of quality of life due to disease-related or treatment-related symptoms, such as seizures and RT-induced neurocognitive deficits, respectively. Therefore, developing novel treatment strategies remains an urgent need, and it is not surprising that *IDH* mutations have become the most investigated therapeutic target in the field of LGGs.

Data from early clinical trials with the first-generation IDH-mut inhibitors in gliomas demonstrated brain penetrance, target engagement, a favorable safety profile, and preliminary efficacy almost exclusively in patients with non-enhancing glioma. The first-in-class, brain-penetrant dual inhibitor of mut-IDH1/2, vorasidenib, showed, in this particular subgroup of patients, an ORR of 18%, with a very promising median PFS of 36.8 months. Conversely, there were no apparent signs of antitumor activity in patients with enhancing tumors. Emerging preclinical evidence provides a potential explanation for this lack of effectiveness against enhancing lesions, suggesting that 2-HG, at least for a subset of gliomas, becomes non-essential for tumor maintenance as they progress to higher-grade tumors, and this transition likely coincides with acquisition of tertiary driver genetic alterations [26,28]. IDH-mut inhibitors would thus exert their full therapeutic potential used early during the natural history of LGGs, before *IDH* mutations convert from a driver to passenger. These observations led to the design of the INDIGO trial, a multicenter, randomized, placebo-controlled Phase III study comparing the efficacy of vorasidenib with placebo in patients with residual/recurrent mut-IDH1/2 grade 2 glioma who have not yet received previous radiation or CT and are under radiographic surveillance following surgery (NCT04164901). The recruitment of this trial is close to being completed, and results will inform us on the ability of IDH-mut inhibitors to prevent the evolution of LGGs towards higher-grade forms, limiting or postponing in this young patient population the use of RT/CT and the onset of their related side effects, such as, primarily, the deterioration of neurocognitive functions. Importantly, we need to clarify which of the 2-HG-induced cellular alterations are reversible thanks to the inhibition of the mut-enzyme. A much deeper understanding of these phenomena also becomes crucial to rationally design combination strategies potentially active, even in cases of acquired resistance. Based on the highly specific and homogenous nature of *IDH* mutations, immunotherapeutic strategies targeting IDH1^R132H^ are currently under clinical evaluation. Initial data from the first-in-human IDH1 mutation-specific peptide vaccination study (NOA16) carried on by the German Group demonstrated the safety, feasibility, and immunogenicity of such approach, with vaccine-related AEs restricted to grade 1 and the vast majority (93.3%) of patients developing effective immune responses across multiple MHC alleles. Moreover, outcomes of patients vaccinated are, despite being preliminary, doubtless encouraging. Besides vaccines, given the highly immunosuppressive glioma microenvironment, immune checkpoint blockade is also being evaluated in many clinical trials, mainly in combinations with other agents. However, mature data defining their potential role in the context of IDH-mut gliomas are still lacking.

On the other hand, mut-IDH also induces a unique pattern of cellular dependencies and weaknesses, which can be exploited as an alternative therapeutic approach. Strategies targeting metabolic deficiencies, the epigenetic profile, or DNA repair pathways’ deficits have proven effective in current preclinical models available, and many of these agents have started their development pathway in the clinic. A particular emphasis, in our opinion, should be placed on the clinical development of PARP inhibitors exploiting the HR DNA repair deficiencies and the “BRCAness” phenotype of the IDH-mut cells, being potentially synergistic with RT, CT, and IDH-mut inhibitors. Results of ongoing clinical trials will hopefully clarify the future direction of IDH-mut glioma treatment.

## Figures and Tables

**Table 1:** Preclinical studies of IDH-targeted agents in glioma mouse models.

Reference	Agents	Results	Additional Findings
Rohle, D. et al., 2013 [27]	AG-5198	50–60% growth inhibition (*p* = 0.015, two-tailed *t*-test) No toxicity along 3 weeks of daily treatment	Reduced staining with Ki-67 antibody in treated mice
Tateishi, K. et al., 2015 [28]	IDH1i	Near-complete elimination of 2-HG within brain tumors after 5 days of treatment No effect on tumor size and survival: mOS 46 days in both treated mice and controls (95% CI; 45–48; *p* = 0.79)	No effect on expression of IDH1, Ki-67, GFAP, or nestin within the tumors
Popovici-Muller, J. et al., 2018 [29]	Ivosidenib (AG-120)	Low brain penetration in rats with intact blood–brain–barrier: 4.1% (AUC 0−8 h (brain)/AUC 0−8 h (plasma)) Robust time-dependent, reversible tumor 2-HG reduction (IC50 range 5–13 nM)	
Pusch, S. et al., 2017 [30]	BAY 1436032	Significantly reduced 2-HG concentration (*p* = 0.00000057) and prolonged survival (*p* = 0.025) compared to untreated controls	SOX2 expression reduced by half in tumors of treated mice
Konteatis, Z. et al., 2020 [31]	Vorasidenib (AG-881)	>97% inhibition of 2-HG production in mice glioma tissue	
Bunse, L. et al., 2018 [32]	BAY 1436032	Oral administration of BAY-1436032 in combination with PD-1 inhibition increased overall survival in mice	Enhanced intratumoral CD4 T-cell proliferation
Schumacher, T. et al., 2014 [33]	IDH1 (R132H) peptide vaccine	Effective control growth in syngeneic IDH1 (R132H)-expressing tumors	Robust interferon IFN-γ T-cell response Mutation-specific anti-IDH1 antibodies detectable in serum of immunized mice
Pellegatta, S. et al., 2015 [34]	IDH1 (R132H) peptide vaccine	Significant survival gain compared to controls with 25% of cured mice	Higher amounts of peripheral CD8+ T-cells, higher production of IFN-γ, anti-IDH1-mut antibodies in immunized mice
Kadiyala, P. et al., 2021 [35]	AGI-5198 +/− IR	2-HG levels in mIDH1 brain tumor tissue reduced by approximately 2.4-fold (*p* ≤ 0.0001) after treatment with AGI-5198 40% long-term survivors among mice treated with AGI-5198 or AGI-5198 + IR Anti-PD-L1 + AGI-5198 + IR and TMZ improved 90-day survival rate by 40% (95% CI: 0–95%, 1-sided *p* = 0.08)	AGI-5198 administration led to a 3-fold (*p* ≤ 0.001) increase in the PD-L1 expression on the CD45–/Nestin+ tumor cells compared with untreated controls Increased infiltration of CD8+ T-cells (*p* < 0.01) in tumors treated with anti-PD-L1 + AGI-5198 + IR and TMZ

2-HG 2-hydroxyglutarate; AUC area under the curve; CD4 cluster of differentiation 4; CD8 cluster of differentiation 8; GFAP glial fibrillary acidic protein; IC50 half maximal inhibitory concentration; IDH isocitrate dehydrogenase; IFN-γ interferon γ; IR ionizing radiation; mOS median overall survival; PD-1 programmed cell death protein 1; PD-L1 programmed death-ligand 1; SOX2 SRY-Box Transcription Factor 2; TMZ temozolomide.

**Table 2:** Clinical trials with IDH-targeted therapies in gliomas with available data.

Reference	NCT Number	Study Design	Treatment	Population	Main Results	Adverse Events (in ≥10% of Patients)
Mellinghoff, I.K. et al., 2020 [40]	NCT02073994	Phase I	Ivosidenib (AG-120) single agent	Advanced IDH1-mut solid tumors 35 non-enhancing recurrent gliomas 31 enhancing recurrent gliomas	500 mg once daily selected for expansion part DCR 88% vs. 45%; median PFS 13.6 vs. 1.4 months in non-enhancing vs. enhancing cohort	No DLT Headache; fatigue; nausea; vomiting; seizure; diarrhea; aphasia; hyperglycemia; neutropenia; depression; hypophosphatemia; paresthesia
Mellinghoff, I.K. et al., 2021 [41]	NCT02481154	Phase I	Vorasidenib (AG-188) single agent	Advanced IDH1 and/or IDH2-mut solid tumors 22 non-enhancing recurrent gliomas 30 enhancing recurrent gliomas	Recommended dose <100 mg in gliomas Non-enhancing glioma: ORR 18% (1 PR; 3 minor responses; 17 SD) Enhancing glioma: ORR 0% (17 SD) Median PFS: 36.8 vs. 3.6 months in non-enhancing vs. enhancing groups	DLT (grade ≥2 ALT/AST increase) in 5 pts at ≥100 mg dose levels Headache; AST/ALT increase; fatigue; nausea; seizure; hyperglicemia; vomiting; constipation; dizziness; neutropenia; cough; diarrhea; aphasia; hypoglycemia
Mellinghoff, I.K. et al., 2019 [42]	NCT03343197	Phase I	Perioperative Ivosidenib (AG-120) (*n* = 13) or vorasidenib (AG-188) (*n* = 14) single agent	Recurrent non-enhancing IDH1^R132H^-mut LGGs undergoing craniotomy	2-HG concentration 92% (ivosidenib) and 92.5% (vorasidenib) lower in resected tumor tissue of treated patients	Diarrhea; constipation; hypocalcemia; nausea; anemia; hyperglicemia; pruritus; headache; fatigue
Wick, A. et al., 2021 [43]	NCT02746081	Phase I	BAY-1436032 single agent	Advanced IDH1^R132X^-mut solid tumors 26 LGG astrocytoma 13 LGG oligodendroglioma 16 GBM	1500 mg twice daily selected for expansion cohorts LGG: ORR 11% (1 CR; 3 PR; 15 SD) GBM: ORR 0%, SD 29%. PFS-rate at three months: 0.31 vs. 0.22 in LGG vs. GBM	No DLT Fatigue; disgeusia
Natsume, A. et al., 2019 [44]	NCT03030066	Phase I	DS-100b single agent	Recurrent/progressive IDH1^R132X^-mut glioma	125–1400 mg twice daily Non-enhancing glioma (*n* = 9): 2 minor responses; 7 SD Enhancing glioma (*n* = 29): 1 CR; 3 PR; 10 SD	DLT (grade 3 WBC decrease) at 1000 mg twice daily Skin hyperpigmentation; diarrhea; pruritus; nausea; rash; headache
Platten, M. et al., 2021 [45]	NCT02454634	Phase I	IDH1-vac single agent	Newly diagnosed IDHR^132H^-mut grade 3 or 4 astrocytomas	93.3% IDH1-vac induced immune response 3-years PFS: 63% 3-years OS: 84%	No RLTs Mild site reactions

2-HG 2-Hydroxyglutarate; ALT alanine transaminase; AST aspartate transaminase; CR complete response; DCR disease control rate; DLT dose-limiting toxicity; GBM glioblastoma; IDH Isocitrate dehydrogenase; LGG low-grade glioma; ORR objective response rate; OS overall survival; PFS progression-free survival; PR partial response; RLT regime-limiting toxicity; SD stable disease; WBC white blood cells.

**Table 3:** Ongoing clinical trials in IDH-mutant gliomas.

NCT Number	Study Phase	Population	Experimental Treatment	Status
NCT04164901	Phase 3	Residual or recurrent IDH1/2-mut grade 2 gliomas	Vorasidenib versus placebo	Recruiting
NCT03684811	Phase 1b/2	Advanced IDH1-mut gliomas and other solid tumors (HCC; bile duct carcinoma; cholangiocarcinoma; other epatobiliary carcinomas; chondrosarcoma)	FT 202 single agent or in combination with chemotherapy (azacitidine; gemcitabine and cisplatin) or immunotherapy (nivolumab)	Active, not recruiting
NCT02968940	Phase 2	IDH-mut GBM	Avelumab and hypofractionated RT	Completed
NCT03991832	Phase 2	Advanced IDH-mut gliomas and other solid tumors (cholangiocarcinoma and others)	Durvalumab and Olaparib	Recruiting
NCT03557359	Phase 2	Recurrent/progressive IDH-mut gliomas	Nivolumab	Active, not recruiting
NCT03718767	Phase 2	IDH-mut gliomas	Nivolumab	Recruiting
NCT03925246	Phase 2	Recurrent IDH-mut high grade gliomas	Nivolumab	Active, not recruiting
NCT03212274	Phase 2	Advanced IDH1/2-mut gliomas and other solid tumors (cholangiocarcinoma and others)	Olaparib	Recruiting
NCT03561870	Phase 2	Recurrent IDH-mut gliomas	Olaparib	Active, not recruiting
NCT03749187	Phase 1	IDH1/2-mut gliomas	PARP inhibitor (BGB-290) and TMZ	Recruiting
NCT03914742	Phase 1/2	IDH1/2-mut gliomas	PARP inhibitor (BGB-290) and TMZ	Recruiting
NCT02702492	Phase 1	Solid tumors or NHL	KPT-9274 (dual inhibitor of PAK4 and NAMPT) ± Nivolumab	Terminated
NCT03666559	Phase 2	Recurrent IDH1/2-mut gliomas	Azacitidine	Recruiting
NCT03922555	Phase 1	Recurrent/progressive non-enhancing IDH-mut gliomas	ASTX727 (cedazuridine + cytidine antimetabolite decitabine)	Recruiting

GBM glioblastoma; HCC hepatocellular carcinoma; IDH isocitrate dehydrogenase; NAMPT nicotinamide phosphoribosyltransferase; PAK4 p21-activated kinase 4; PARP poly (ADP-ribose) polymerase.
